# Supplementary material for: Probing multi-spinon excitations outside of the two-spinon continuum in the antiferromagnetic spin chain cuprate Sr2CuO3
Source: Nat Commun. 2018 Dec 19;9:5394. doi: 10.1038/s41467-018-07838-y (PMC6300594; doi:10.1038/s41467-018-07838-y)
Supplement: Supplementary file 1 — Supplementary Information [file 41467_2018_7838_MOESM1_ESM.pdf]

Supplementary Material for:

Probing multi-spinon excitations outside of the two-spinon continuum in the antiferromagnetic spin chain cuprate Sr_2CuO_3

Schlappa et al.

Supplementary Note 1: Experimental geometry

A sketch of the scattering geometry is given in Supplementary Figure 1**a**. Supplementary Figure 1**b** shows the corresponding mapping between the incident angle and momentum transfer along the chain direction (x-axis) in O *K*-edge RIXS, as well as the polarization dependence for RIXS scattering intensity, related to excitations within the Lower Hubbard Band (LHB), which involve plaquette-connecting oxygen sites.

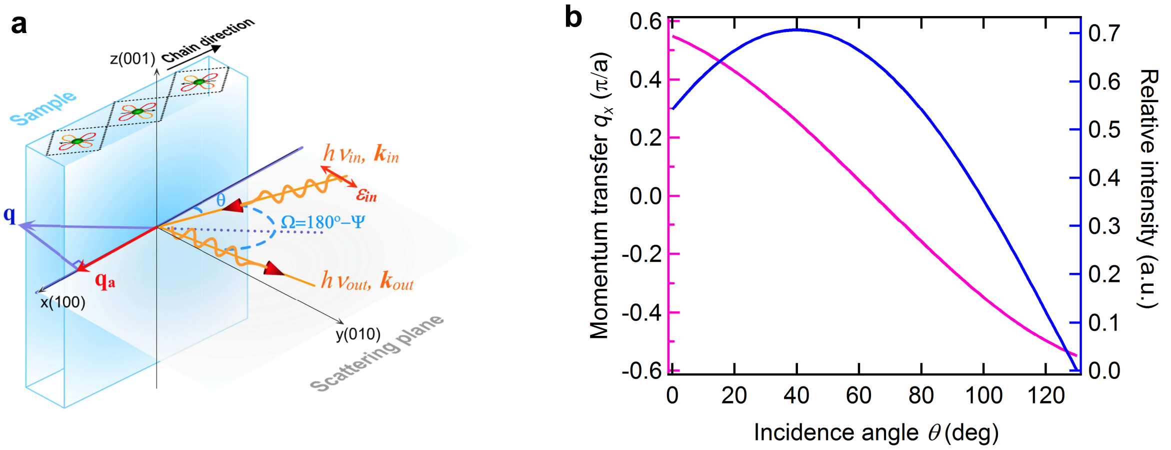

Supplementary Figure 1: A schematic of the experimental geometry. Panel **a** shows the scattering geometry. CuO_4 plaquettes of the 1D chain lie in the scattering plane. The incidence angle, θ, is varied during the experiment to change the projection of the momentum transfer along the chain direction ($\mathbf{e}_x$), whereas the scattering angle $\psi = 130°$ is kept constant. Panel **b** shows the momentum transferred along the chains, q_x, as function of θ (magenta solid line) – as well as the polarization dependence of the scattering intensity (blue solid line) related to intra-LHB RIXS excitations of in-chain oxygens.

Supplementary Note 2: RIXS spectra in the full Brillouin zone

Due to the relative low energy of x-ray photons used for the excitations at the oxygen K-edge, the technique can only access about half of the first Brillouin zone (BZ). One can, however, measure out to the Brillouin zone boundary at other elemental edges such as the Cu $L_{2,3}$-edge. Similarly, we can also compute the RIXS spectra throughout the entire BZ. By examining the magnetic excitations throughout the 1st BZ, we can gain further insight into their identities. Supplementary Figure 2 compares the measured and calculated RIXS spectra over the available momentum-transfer range: O *K*-edge data (Supplementary Figure 2**a**), theory (Supplementary Figure 2**b**), Cu L_3-edge data (Supplementary Figure 2**c**). One can see that the calculated weight of the dispersing branch agrees well with the continuum observed at the Cu L_3-edge (primarily due to two-spinon excitations) out to the zone boundary.

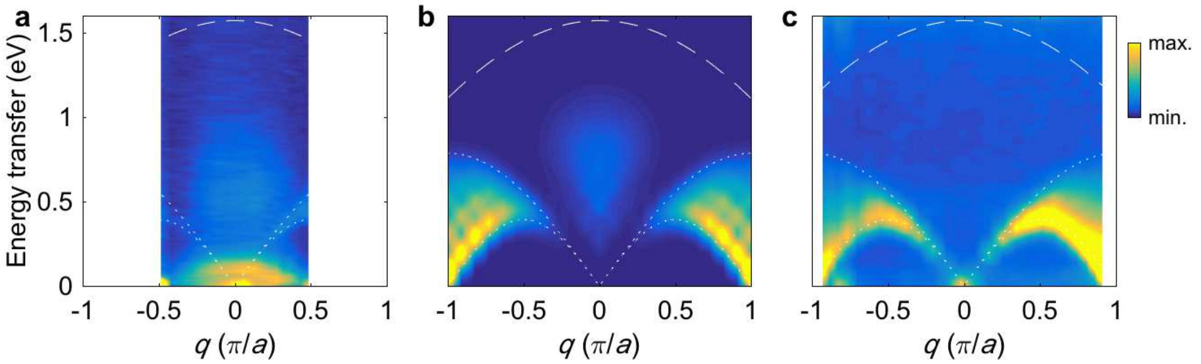

Supplementary Figure 2: RIXS spectra plotted over the full first Brillouin zone. a The experimental spectra at the O *K*-edge shown in the momentum range accessible to the experiment. **b** The calculated RIXS spectra at O *K*-edge over the entire 1st BZ. **c** the experimental data at Cu L_3-edge. The dotted and dashed lines overlaid over the data shows the boundaries for the two- and four-spinon continuua.

Supplementary Note 3: Low-energy excitations and background in O *K*- and Cu L_3-edge line spectra at the Γ-point

Supplementary Figure 3 shows zoom into O *K*- and Cu L_3-edge line spectra measured at $q = 0$. The data are normalized to acquisition time. The low-energy excitations below 1 eV can be clearly seen in the O *K*-edge spectrum. They are well separated from *dd*-excitations and there is little background in the region 1.2 – 1.6 eV. In contrast, the Cu L_3-edge spectrum has a high background in the low-energy region, which extends from the very strong *dd*-excitations (see Fig. 1 and Supplementary Reference 1) all the way down to the elastic peak. This makes it much more difficult to study excitations of weak spectral weight at the Cu L_3-edge, such as four-spinon excitations. The statistics of the presented O *K*-edge data is better, due to the four times longer acquisition time. The combined energy resolution of the O *K*-edge data is factor 1.8 better (see Methods section).

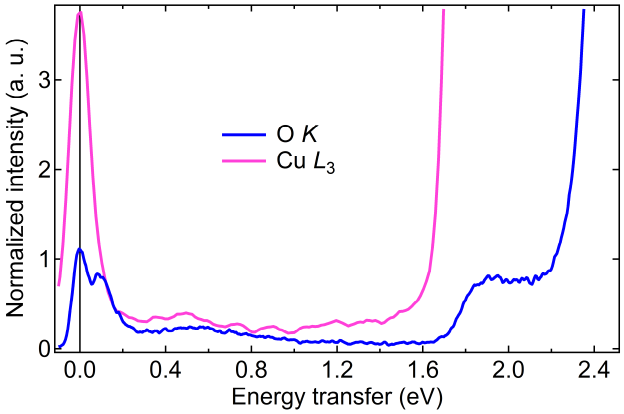

Supplementary Figure 3: The RIXS line spectra measured at the O *K*- and Cu L_3-edges close to the Γ-point ($q = 0$). The O *K*-edge (Cu L_3-edge) spectrum is represented by the blue (magenta) line. The Cu L_3 spectrum has been offset in the vertical by 0.1 for clarity.

Supplementary Note 4: Boundaries of the two- and four-spinon continua

The dispersion of a single spinon is given by $\omega_{\mathrm{s}}(k) = \frac{\pi J}{2}|\sin(ka)|$ and the boundaries of the two-spinon continuum is given by a simple convolution of $\omega_{\mathrm{s}}(k)$ with itself. Similarly, the convolution of four single spinon dispersions will produce the four-spinon continuum. The upper and lower boundaries of the two-spinon continuum are given by $\omega_{2\mathrm{s}}^{\mathrm{u}} = \pi J|\sin(qa/2)|$ and $\omega_{2\mathrm{s}}^{\mathrm{l}} = \frac{\pi J}{2}|\sin(qa)|$. Similarly, the upper and lower boundary of the four-spinon continuum are given by $\omega_{4\mathrm{s}}^{\mathrm{u}} = \sqrt{2}\pi J\left[1+\cos\left(\frac{qa}{2}\right)\right]^{1/2}$ and $\omega_{4\mathrm{s}}^{\mathrm{l}}(q) = \frac{\pi J}{2}|\sin(qa)|$, respectively. These expressions are derived in Supplementary Reference 2 and are determined purely by kinematic constraints. The boundaries of the two- and four-spinon continua are plotted in Supplementary Figure 4.

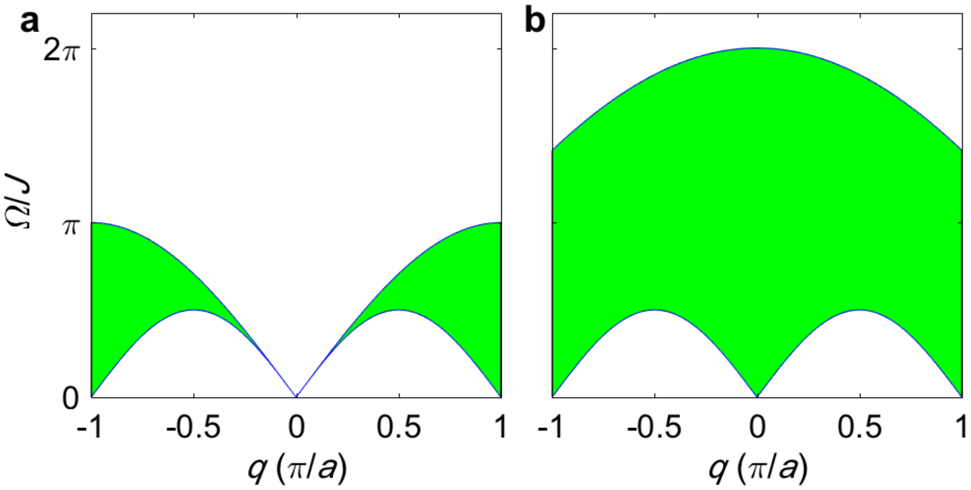

Supplementary Figure 4: The phase space of the two- and four-spinon continuum. The boundaries are derived from the dispersion relationship of non-interacting spinons. Panel **a** shows the two-spinon continuum while panel **b** shows the boundaries of the four-spinon continuum.

Supplementary Note 5: Incidence energy dependence

Supplementary Figure 5 shows how the various magnetic excitations depend on the incident photon energy in our model. The multi-spinon excitations that are prominent at $q = 0$ are very sensitive to the incident energies compared to the dispersing two-spinon excitations, and the multi-spinon excitations have the largest spectral weight for ω_{in} in the range of 0.3 eV to 0.6 eV. This sensitivity to the incident photon energy indicates that the multi-spinon excitations found outside of the two-spinon continuum are more effectively reached via particular intermediate states.

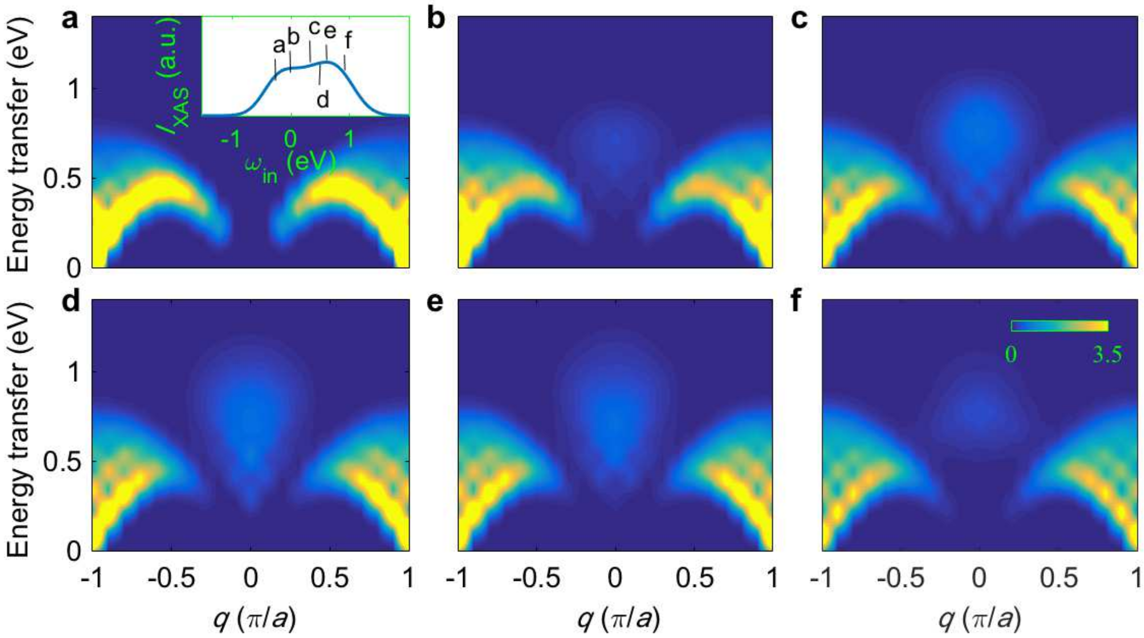

Supplementary Figure 5: RIXS spectra dependence on the incidence energy. Panels **a** through **f** show the RIXS spectra evaluated at various incident energy as indicated in XAS spectra shown in the inset of panel **a**.

Supplementary References

1. J. Schlappa, K. Wohlfeld, K. J. Zhou, M. Mourigal, M. W. Haverkort, V. N. Strocov, L. Hozoi, C. Monney, S. Nishimoto, S. Singh, A. Revcolevschi, J.-S. Caux, L. Patthey, H. M. Rønnow, J. van den Brink, and T. Schmitt, Spin-orbit separation in the quasi-one-dimensional Mott insulator Sr_2CuO_3. *Nature* **485**, 82 - 85 (2012).
2. J.-S. Caux & Hagemans, The four-spinon dynamical structure factor of the Heisenberg chain. *Journal of Statistical Mechanics: Theory and Experiment,* P12013 (2006).
